# Aging‐Related Myocardial Susceptibility Lowers Cardiac Tolerance to Chronic Intermittent Hypoxia Through Dynamin‐Related Protein 1‐Associated Mitochondrial Vulnerability

**DOI:** 10.1111/acel.70684

**Published:** 2026-08-25

**Authors:** Yinghui Su, Yuyang Miao, Jin Tan, Feng Wang, Qiang Zhang

**Affiliations:** ^1^ Department of Geriatrics Tianjin Medical University General Hospital, Key Laboratory of Post‐Neuroinjury Neuro‐Repair and Regeneration in the Central Nervous System, Ministry of Education, State Key Laboratory of Experimental Hematology, Tianjin Key Laboratory of Elderly Health, Tianjin Geriatrics Institute Tianjin People's Republic of China; ^2^ Department of Genetics, School of Basic Medical Science Tianjin Medical University, The Province and Ministry Co‐Sponsored Collaborative Innovation Center for Medical Epigenetics Tianjin People's Republic of China

**Keywords:** cardiomyocyte, chronic intermittent hypoxia (CIH), dynamin‐related protein 1 (Drp1), mitochondria, myocardium, senescence

## Abstract

Chronic intermittent hypoxia (CIH), a cardinal pathophysiological feature of obstructive sleep apnea (OSA), repeatedly exposes the heart to hypoxia‐reoxygenation stress. The cardiac outcome of CIH, however, may depend on the biological state of the target myocardium. Here, we investigated whether a pre‐existing aging‐related myocardial susceptibility lowers the tolerance threshold for CIH‐induced injury and whether Dynamin‐related protein 1 (Drp1) contributes to the enhanced vulnerability of senescence‐like cardiomyocytes under CIH. Using G3 Tert‐deficient (Tert^−/−^) mice and D‐galactose (D‐gal)‐induced senescence‐like primary cardiomyocytes, we show that aging‐related susceptibility consistently amplifies CIH‐induced cardiac injury. In young wild‐type mice, 8 weeks CIH induced early cardiac remodeling and senescence‐associated myocardial stress without overt systolic decompensation. In Tert^−/−^ mice, the same CIH exposure shifted the cardiac response further toward maladaptive remodeling. Compared with CIH alone, EF and FS were reduced by an additional 24.6% and 15.8%, respectively. In senescence‐like cardiomyocytes, CIH amplified mitochondrial vulnerability, with impaired energy production, elevated mitochondrial oxidative stress, and a fission‐biased mitochondrial dynamics marker profile. Drp1 knockdown did not fully reverse this mitochondrial state but restored 55.1% of the CIH + D‐gal induced ATP decline and reversed 47.4% of the mitochondrial ROS excess, while attenuating DNA damage response activation and senescence‐associated signaling. These findings indicate that aging‐related myocardial susceptibility is not a passive contextual factor in CIH‐induced injury but a biological state that actively shapes the cardiac response to repeated hypoxia‐reoxygenation stress. Drp1‐associated mitochondrial dynamics imbalance may represent a functional link between diminished mitochondrial stress tolerance and amplified cardiomyocyte vulnerability.

## Graphical abstract

Chronic intermittent hypoxia (CIH) induces early myocardial remodeling and senescence‐associated injury without overt systolic failure in young mice. Aging‐related susceptibility, modeled in vivo by Tert^−/−^ mice and in vitro by D‐gal‐treated primary cardiomyocytes, amplifies CIH‐associated cardiac dysfunction and cellular injury. Drp1 knockdown partially attenuates mitochondrial dysfunction, DNA damage response activation, and senescence‐associated phenotypes, supporting a functional contribution of Drp1 to reduced CIH tolerance.

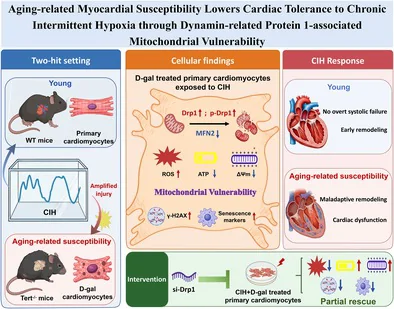

## 1 Introduction

Obstructive sleep apnea (OSA) is a highly prevalent sleep‐related breathing disorder, particularly common in middle‐aged and older populations, and is closely associated with an increased risk of cardiovascular disease (Benjafield et al. 2019; Iannella et al. 2025; Yeghiazarians et al. 2021). Chronic intermittent hypoxia (CIH) is one of the central pathophysiological features of OSA. Repeated hypoxia‐reoxygenation cycles during sleep expose the heart to multiple stress conditions, including oxidative stress, inflammation, sympathetic activation, and metabolic disturbance (Gottlieb and Punjabi 2020; Javaheri, Javaheri, Somers, et al. 2024; Tasali et al. 2025). Previous clinical and experimental studies have linked OSA or CIH exposure to myocardial injury, cardiac remodeling, arrhythmia, and impaired cardiac function (Yeghiazarians et al. 2021; Redline et al. 2023; Javaheri, Javaheri, Gozal, et al. 2024). However, CIH‐associated cardiac injury is unlikely to be determined solely by hypoxic burden. The tolerance of the myocardium to repeated hypoxia‐reoxygenation may differ across biological backgrounds, suggesting that the intrinsic state of the target myocardium may be an important determinant of the cardiac outcome after CIH exposure.

Aging is an important biological context that shapes cardiac stress tolerance, but it is not equivalent to chronological age itself. The myocardium in an aging‐related state is often characterized by reduced mitochondrial reserve, impaired redox homeostasis, enhanced DNA damage response, disrupted proteostasis, and accumulation of senescence‐associated molecular signals (Chen et al. 2022; Ali et al. 2023; Miwa et al. 2022; Mainali et al. 2023; Anderson et al. 2019). These alterations do not necessarily cause overt cardiac decompensation under baseline conditions but may weaken the ability of the myocardium to maintain homeostasis under additional stress. It remains unclear whether an aging‐related susceptible state makes the myocardium more vulnerable to repeated hypoxia‐reoxygenation stress, thereby promoting maladaptive injury rather than compensatory adaptation.

Mitochondrial dysfunction may represent an important point of convergence between aging‐related myocardial susceptibility and CIH stress. Cardiomyocyte energy metabolism and redox homeostasis are highly dependent on mitochondrial function (Kolwicz et al. 2013; Bertero et al. 2016). Repeated hypoxia‐reoxygenation can disturb mitochondrial electron transport and ROS generation (Khan et al. 2010; Korge et al. 2008), whereas aging‐related states are often accompanied by reduced mitochondrial adaptive capacity (Lesnefsky et al. 2016). Mitochondrial dynamics determine mitochondrial network integrity and stress adaptability, and Dynamin‐related protein 1 (Drp1) is an important regulator of mitochondrial fission and mitochondrial network remodeling (Eisner et al. 2018; Tábara et al. 2024; Ong et al. 2012). However, whether Drp1‐related alterations in mitochondrial dynamics contribute to the amplification of CIH‐induced injury in an aging‐related susceptible myocardial state remains unclear.

Based on this rationale, the present study aimed to determine whether a pre‐existing aging‐related susceptible myocardial background modifies the cardiac response to CIH, and to further evaluate the role of Drp1‐dependent mitochondrial dynamics in this process. We combined an in vivo Tert‐deficient mouse model (Tert^−/−^) with an in vitro D‐galactose (D‐gal)‐induced senescence‐like primary cardiomyocyte model to assess CIH‐induced myocardial remodeling, senescence‐associated injury and mitochondrial stress responses. In primary cardiomyocytes, siRNA‐mediated Drp1 knockdown was further used to examine its functional contribution. We hypothesized that an aging‐related susceptible background may reduce myocardial tolerance to repeated hypoxia‐reoxygenation stress, and that altered mitochondrial dynamics may contribute to the enhanced susceptibility of senescence‐like cardiomyocytes to CIH‐induced injury. A list of abbreviations used in this manuscript is provided in Table S2.

## 2 Methods and Materials

### 2.1 Animals and Ethical Approval

We used two strains of female mice for this study: wild‐type C57BL/6J mice (8–10 weeks old; Chinese Academy of Military Sciences, Beijing, China) and G3 Tert^−/−^ mice (8–10 weeks old; generously provided by Professor Feng Wang, Tianjin Medical University) (Wang et al. 2026). The Tert^−/−^ strain was generated by crossing Tert^+/−^ female mice with Tert^+/−^ male mice. Wild type C57BL/6J mice were used as non‐littermate controls. A specific‐pathogen‐free (SPF) barrier facility served as the housing environment for all mice, with unrestricted access to sterilized water and standard laboratory diet. All animal experimental protocols were approved by the Institutional Animal Care and Use Committee (IACUC) of Tianjin Medical University. Throughout the study, all animal procedures were performed in strict accordance with the National Institutes of Health Guide for the Care and Use of Laboratory Animals.

### 2.2 CIH Exposure in Mice

For CIH exposure, mice were maintained in an intermittent hypoxia chamber with the fraction of inspired oxygen (FiO_2_) circulating between 21% and 5% under standardized conditions (22°C–24°C; < 50% humidity) (Kazim et al. 2021; Khalyfa et al. 2021). The mice were exposed to CIH for 8 h daily at a rate of 40 cycles/h for 8 weeks. Normoxic controls were subjected to ambient atmospheric conditions (21% O_2_) for the same duration.

### 2.3 Experimental Design, Randomization, and Blinding

Two independent mouse cohorts were used. The young wild‐type cohort consisted of C57BL/6J mice allocated to normoxic control or CIH exposure conditions. For the genotype‐based cohort, mice were divided into four groups: Control, CIH, Tert^−/−^, and CIH + Tert^−/−^. Mice from the same genotype were randomly allocated to normoxic or CIH exposure conditions. Sample sizes were based on previous CIH‐related studies, experimental feasibility, and the availability of Tert^−/−^ mice. All outcome analyses were performed using coded sample identifiers, so the investigators were blinded to group allocation during data analysis.

### 2.4 Echocardiography

In the young wild‐type cohort, three mice were randomly selected from the CIH group before exposure and individually marked. The same mice underwent serial transthoracic echocardiography at baseline and after 2, 4, and 8 weeks of CIH exposure to characterize temporal changes in cardiac function. Serial imaging was restricted to this subset to limit the number of animals subjected to repeated anesthesia and handling, consistent with the 3Rs principle; no other animals in this cohort underwent echocardiographic assessment. In the genotype‐based cohort, five mice were randomly selected from each group for echocardiographic assessment at the 8‐week endpoint.

Echocardiography was performed under anesthesia with 1.5% isoflurane (RWD Life Science, China). Briefly, mice were placed in a supine position after removal of the precordial fur, and images were obtained in the parasternal short‐axis view using a Vevo 3100LT system (FUJIFILM VisualSonics, Tokyo, Japan). Fractional shortening (FS) and left ventricular ejection fraction (EF) were analyzed using M‐mode tracings. To preserve physiological consistency during acquisition, heart rates were maintained within a physiological range of 400–500 beats per minute (bpm).

### 2.5 Serological Biochemical Parameters

Mice were fasted for 12 h before retro‐orbital blood samples were collected under isoflurane anesthesia. Serum was obtained by centrifuging coagulated blood at 1000 × *g* for 30 min at room temperature. Serum was analyzed for creatine kinase (CK) and lactate dehydrogenase (LDH) using commercial assay kits (Beyotime, CK, S0287S; LDH, P0393S) according to the manufacturers' instructions.

### 2.6 Tissue Histopathology

At the end of the 8‐week CIH protocol, hearts were collected after euthanasia under anesthesia, washed with cold PBS, and fixed overnight in 4% paraformaldehyde (PFA), embedded in paraffin, and sectioned at 5 μm. Cardiac sections were processed using standard hematoxylin and eosin (H&E; Solarbio, G1125, Beijing, China) protocols for general cardiac morphology and with the Masson trichrome staining (Solarbio, G1346, Beijing, China) for collagen deposition.

### 2.7 Isolation and Culture of Neonatal Mouse Primary Cardiomyocytes

Neonatal mouse primary cardiomyocytes were isolated from 1 to 3‐day‐old C57BL/6J mice. Briefly, hearts were rapidly excised, washed with ice‐cold PBS, and minced into 0.5–1 mm^3^ fragments. The tissue fragments were first incubated with 0.0625% trypsin (Solarbio, T1300, Beijing, China) for 5 min at room temperature, and the supernatant was discarded. The remaining tissue was then digested repeatedly with 0.1% collagenase II (Solarbio, C8150, Beijing, China) at 37°C for 5 min per cycle until the tissue became nearly translucent. The collected cell suspensions were neutralized with DMEM/F12 containing 15% fetal bovine serum (FBS), filtered through a 70 μm cell strainer, and centrifuged at 1200 rpm for 10 min at room temperature. The cell pellet was resuspended in culture medium and pre‐plated in T25 flasks for 60 min to reduce fibroblast contamination. Non‐adherent cells were collected, counted using trypan blue dye, and seeded onto plates pre‐coated with 0.1% gelatin (Solarbio, G0040, Beijing, China) for subsequent experiments. BrdU (MedChemExpress, HY‐15910, NJ, USA) was added to the culture medium at a final concentration of 0.1 mM to inhibit fibroblast proliferation. Cells were maintained at 37°C in a humidified incubator with 5% CO_2_.

### 2.8 Assessment of Primary Cardiomyocyte Purity

Primary cardiomyocyte purity was assessed 48 h after seeding by immunofluorescence staining for cTnT and DAPI. The purity was calculated as the percentage of cTnT‐positive cells among total DAPI‐positive cells.

### 2.9 D‐Gal and CIH Treatment in Primary Cardiomyocytes

After purity assessment, primary cardiomyocytes were assigned to four groups: Control, CIH, D‐gal, and CIH + D‐gal. Cells in the D‐gal and CIH + D‐gal groups were treated with D‐gal (20 g/L) for 36 h. Cells in the CIH and CIH + D‐gal groups were then exposed to CIH for 12 h, while control cells were maintained under normoxic conditions for the same duration. D‐gal remained present in the culture medium during CIH exposure in the CIH + D‐gal group.

### 2.10 siRNA‐Mediated Drp1 Knockdown

Primary cardiomyocytes were transfected with si‐Drp1 or si‐NC using CALNP RNAi in vitro transfection reagent (D‐Nano Therapeutics, Beijing, China). The siRNA transfection mixture was prepared according to the manufacturer's instructions and added to 6‐well plates at a final siRNA concentration of 30 nM. The medium was replaced with the corresponding treatment medium 24 h after transfection. Cells were assigned to three groups: si‐NC, CIH + D‐gal + si‐NC, and CIH + D‐gal + si‐Drp1. Drp1 knockdown efficiency was confirmed by Western blotting before subsequent functional experiments.

### 2.11 Senescence‐Associated Beta‐Galactosidase (SA‐*β*‐Gal) Detection

For cardiac tissues, frozen sections were incubated with YDGAL working solution for 1 h at room temperature in the dark, counterstained with DAPI, and imaged using an Olympus fluorescence microscope (Heidelberg, Germany). SA‐*β*‐gal activity in myocardial tissue was assessed by quantifying YDGAL fluorescence intensity.

For primary cardiomyocytes, cells were fixed and incubated with SA‐*β*‐gal staining solution (Solarbio, G1580, Beijing, China) overnight at 37°C. After washing, cells were imaged using an Olympus optical microscope (Heidelberg, Germany), and the SA‐*β*‐gal‐positive rate was calculated as the percentage of blue‐stained cells among total cells.

### 2.12 DHE Staining in Cardiac Tissues

Mouse heart cryosections were incubated with 5 μM dihydroethidium (DHE) for 30 min at 37°C in the dark. After washing, sections were counterstained with DAPI. DHE fluorescence intensity was quantified to assess superoxide production in cardiac tissues.

### 2.13 Assessment of Mitochondrial ROS and Mitochondrial Membrane Potential

Mitochondrial ROS levels were detected using MitoSOX Red (Invitrogen, M36008, USA). Primary cardiomyocytes were incubated with 5 μM MitoSOX Red for 10 min at 37°C in the dark, washed with prewarmed PBS, and counterstained with Hoechst 33,342 for fluorescence imaging. Mitochondrial membrane potential was assessed using a JC‐1 assay kit (Beyotime, C2003S, China). Cells were incubated with JC‐1 working solution for 20 min at 37°C in the dark and washed with JC‐1 buffer. Fluorescence images were acquired using an Olympus fluorescence microscope (Olympus, Heidelberg, Germany). MitoSOX fluorescence intensity and the JC‐1 red to green fluorescence ratio were quantified using ImageJ software. For flow cytometry, stained cells were collected, washed, resuspended in PBS, and analyzed immediately. MitoSOX mean fluorescence intensity and the JC‐1 red to green fluorescence ratio were quantified using FlowJo software (vX.0.7, FlowJo LLC, USA).

### 2.14 ATP Measurement

ATP levels in primary cardiomyocytes were measured using an ATP assay kit based on the phosphomolybdic acid colorimetric method (Puxi Biotechnology, PB1199W48, China) according to the manufacturer's instructions. Absorbance was measured using a microplate reader, and ATP levels were normalized to total protein concentration and expressed relative to the control group.

### 2.15 Immunofluorescence (IF)

Primary cardiomyocytes were fixed with 4% PFA. For cardiac cryosections, mice were euthanized under anesthesia and perfused with cold PBS. Myocardial tissues were fixed in 4% PFA at 4°C overnight, dehydrated in 15% and 30% sucrose, embedded in optimal cutting temperature (OCT) compound (Sakura, Torrance, CA, USA), and cryosectioned. Cells and cardiac sections were washed, permeabilized, and blocked with 3% BSA for 1 h. Samples were incubated with primary antibodies listed in Table S1 at 4°C overnight, followed by fluorophore‐conjugated secondary antibodies for 1 h at room temperature. Nuclei were counterstained with DAPI. Images were captured using an Olympus fluorescence microscope (Olympus, Heidelberg, Germany).

### 2.16 Immunoblotting

Protein lysates from mouse hearts and primary cardiomyocytes were mixed with loading buffer and denatured at 100°C for 10 min. Equal amounts of protein were separated by 10%–12% SDS‐PAGE and transferred onto PVDF membranes. Membranes were blocked with 5% skim milk for 2 h at room temperature and incubated with primary antibodies listed in Table S1 at 4°C overnight. After washing with TBST, membranes were incubated with HRP‐conjugated secondary antibodies, followed by chemiluminescent detection. Protein signals were visualized using a Bio‐Rad imaging system, and band intensities were quantified using ImageJ software.

### 2.17 Statistical Analysis

Statistical analysis was performed using GraphPad Prism 9.0 (GraphPad Software, San Diego, CA, USA). Data are presented as mean ± SEM with individual data points. Comparisons between two groups were analyzed using an unpaired two‐tailed Student's *t*‐test. Multiple‐group comparisons were analyzed using one‐way ANOVA, whereas longitudinal echocardiographic data were analyzed using one‐way repeated‐measures ANOVA. Tukey's multiple‐comparisons test was used for post hoc comparisons. A *p*‐value < 0.05 was considered statistically significant.

## 3 Results

### 3.1 CIH Induces Early Cardiac Remodeling and Senescence‐Associated Myocardial Injury in Young Wild‐Type Mice

**FIGURE 1:**
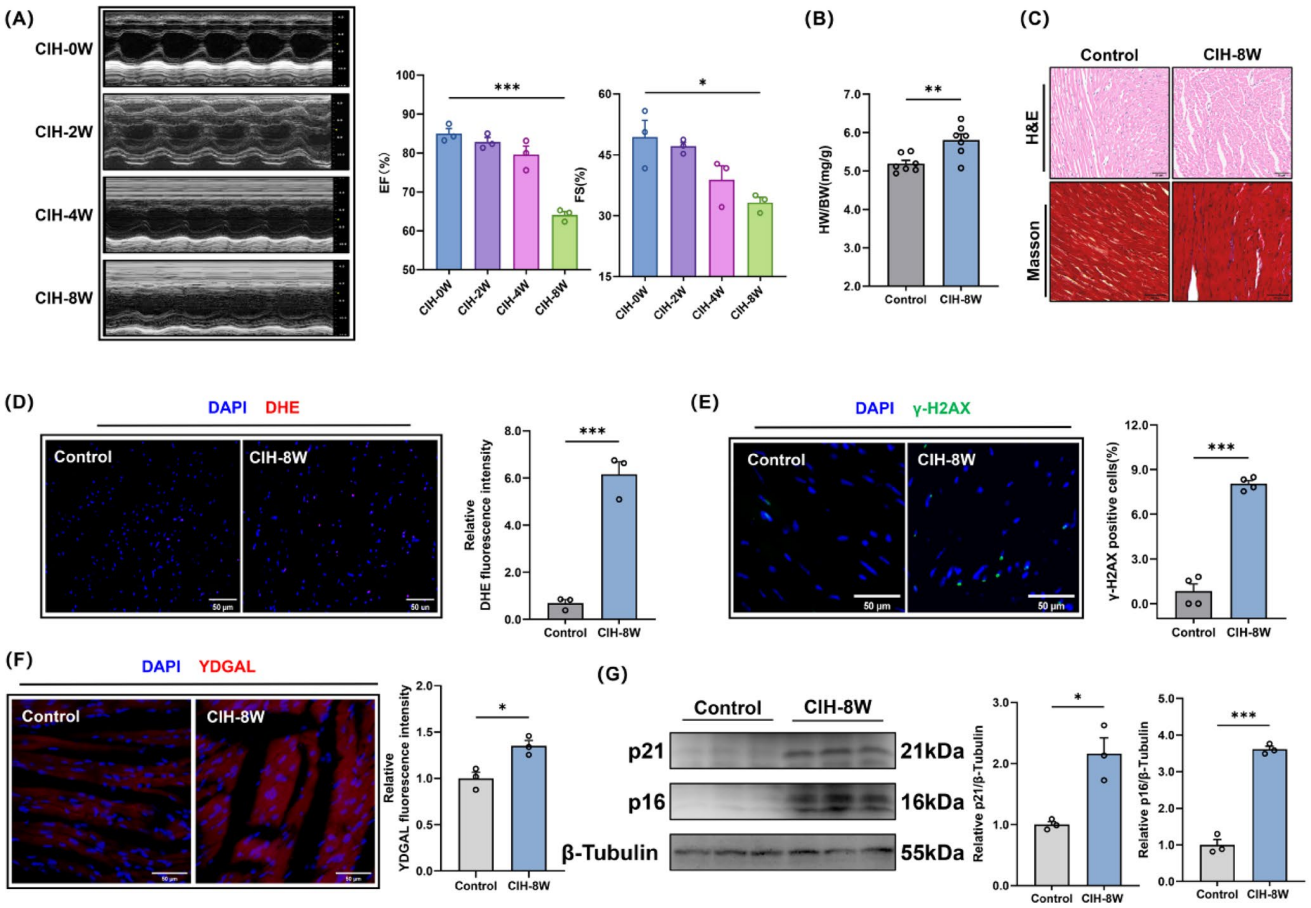
CIH induces early cardiac remodeling, oxidative stress, DNA damage response and senescence‐associated myocardial injury in young mice. (A) Representative M‐mode echocardiograms and quantitative analysis of EF and FS from three mice randomly selected from the CIH group and followed at baseline and after 2, 4, and 8 weeks of CIH exposure (*n* = 3). Values for each mouse were averaged from 3 to 5 consecutive cardiac cycles. The adjusted *p* values for baseline versus 8 weeks were < 0.001 for EF and 0.015 for FS. (B) Heart weight to body weight ratio in control and CIH‐exposed mice after 8 weeks of exposure (*n* = 7/group). (C) Representative H&E and Masson trichrome staining of cardiac sections from control and CIH‐exposed mice after 8 weeks of exposure. Scale bar = 50 μm. (D) Representative DHE staining for myocardial ROS production and quantification of relative DHE fluorescence intensity (*n* = 3/group; red, DHE; blue, DAPI; scale bar = 50 μm). (E) Representative *γ*‐H2AX immunofluorescence staining and quantification of *γ*‐H2AX‐positive nuclei (*n* = 4/group; green, *γ*‐H2AX; blue, DAPI; scale bar = 50 μm). (F) Representative YDGAL fluorescence images for SA‐*β*‐gal activity and quantification of relative YDGAL fluorescence intensity (*n* = 3/group; red, YDGAL; blue, DAPI; scale bar = 50 μm). (G) Representative Western blots and quantitative analysis of *p*21 and *p*16 protein expression in cardiac tissue (*n* = 3/group). *β*‐Tubulin was used as the loading control. Western blot densitometry and fluorescence intensity values were normalized to the corresponding control group. Data are presented as mean ± SEM and analyzed using one‐way repeated‐measures ANOVA or unpaired two‐tailed Student's *t*‐test, as appropriate. *p < 0.05, **p < 0.01, ***p < 0.001.

To determine whether prolonged CIH exposure is sufficient to induce cardiac injury in a non‐aged background, young wild‐type C57BL/6J mice were exposed to CIH for up to 8 weeks. Serial echocardiography in the randomly selected CIH subset (*n* = 3) showed a progressive decline in left ventricular systolic indices during exposure. EF and FS began to decrease after 4 weeks of CIH exposure and showed a greater reduction at 8 weeks (Figure 1A). Therefore, the 8‐week time point was selected for subsequent histological and molecular analyses.

After 8 weeks of CIH exposure, the heart weight to body weight ratio (HW/BW) was higher in CIH‐treated mice than in normoxic controls (Figure 1B). H and E staining showed myocardial fiber disarray in CIH‐exposed hearts, and representative Masson trichrome staining suggested more prominent interstitial collagen staining after CIH exposure (Figure 1C). These findings suggest that 8 weeks of CIH exposure induces early myocardial injury and structural remodeling, but does not constitute an overt heart failure phenotype.

Because CIH consists of repeated hypoxia‐reoxygenation cycles, oxidative stress is considered an early event in CIH‐induced myocardial injury. Sustained ROS accumulation can also activate DNA damage response and promote senescence‐associated molecular programs. Therefore, we next examined whether CIH‐induced cardiac remodeling was accompanied by oxidative stress, DNA damage response, and senescence‐associated molecular changes. DHE staining showed increased myocardial ROS generation after 8 weeks of CIH exposure (Figure 1D). CIH also increased the percentage of *γ*‐H2AX‐positive nuclei, indicating activation of a DNA damage response (Figure 1E). YDGAL fluorescence showed increased SA‐*β*‐gal activity in CIH‐exposed myocardium (Figure 1F). In parallel, Western blot analysis showed upregulation of the senescence‐associated cell‐cycle inhibitors p21 and p16 in CIH‐exposed hearts compared with controls (Figure 1G). Together, these data indicate that prolonged CIH exposure induces early cardiac remodeling in young wild‐type mice, accompanied by oxidative stress, DNA damage response activation, and senescence‐associated myocardial injury.

### 3.2 A Telomere Dysfunction Background Aggravates Cardiac Remodeling and Senescence‐Associated Cardiomyocyte Injury Under CIH Exposure

**FIGURE 2:**
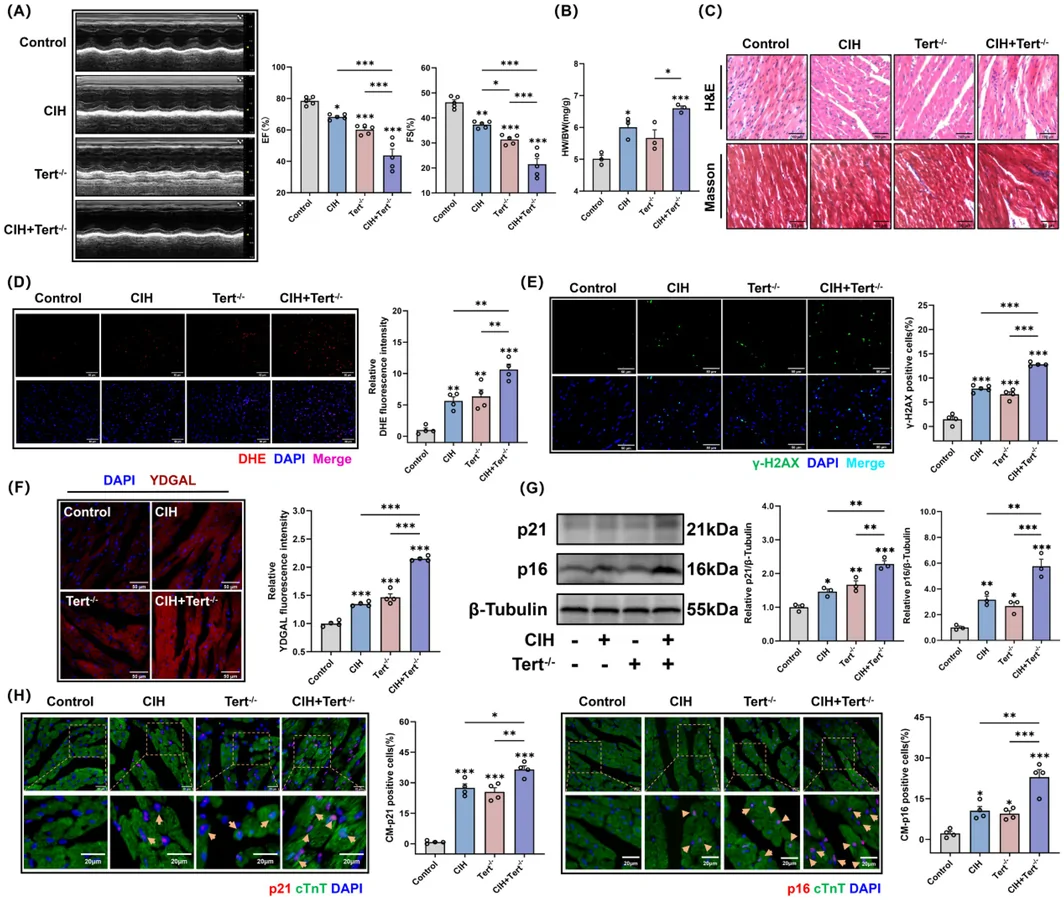
A telomere dysfunction background aggravates CIH‐induced cardiac remodeling, oxidative stress, DNA damage response and senescence‐associated cardiomyocyte injury. Mice were studied in four groups: Control, CIH, Tert^−/−^ and CIH + Tert^−/−^, with all analyses performed after 8 weeks of CIH exposure. (A) Representative M‐mode echocardiograms and quantitative analysis of EF and FS in the four groups. For both EF and FS, the adjusted *p* values were < 0.001 for CIH + Tert^−/−^ versus CIH and versus Tert^−/−^. (B) Heart weight to body weight ratio in the four groups. (C) Representative H&E and Masson trichrome staining of cardiac sections from the four groups (Scale bar = 50 μm). (D) Representative DHE staining for myocardial ROS production and quantification of relative DHE fluorescence intensity (Red, DHE; Blue, DAPI; Scale bar = 50 μm). (E) Representative *γ*‐H2AX immunofluorescence staining and quantification of *γ*‐H2AX‐positive nuclei (Green, *γ*‐H2AX; Blue, DAPI; Scale bar = 50 μm). (F) Representative YDGAL fluorescence images for myocardial SA‐*β*‐gal activity and quantification of relative YDGAL fluorescence intensity (Red, YDGAL; Blue, DAPI; Scale bar = 50 μm). (G) Representative Western blots and quantitative analysis of *p*21 and *p*16 protein expression in cardiac tissue. *β*‐Tubulin was used as the loading control. (H) Representative co‐staining images of cTnT with *p*21 or *p*16, and quantification of cTnT‐positive cardiomyocytes expressing *p*21 or *p*16 (Green, cTnT; Red, *p*21 or *p*16; Blue, DAPI; Scale bar = 20 μm). Sample sizes were as follows: EF and FS, *n* = 5/group; HW/BW, *n* = 3/group; H and E and Masson staining, representative images from *n* = 3 to 4/group; DHE, *γ*‐H2AX, YDGAL and cTnT co‐staining analyses, *n* = 4/group; Western blot analysis, *n* = 3/group. Western blot densitometry and fluorescence intensity values were normalized to the corresponding control group. Data are presented as mean ± SEM and analyzed using one‐way ANOVA. *p < 0.05, **p < 0.01, ***p < 0.001.

To assess whether a senescence‐prone background increases myocardial susceptibility to CIH, we used Tert^−/−^ mice as a telomere dysfunction‐associated model and compared four groups: Control, CIH, Tert^−/−^, and CIH + Tert^−/−^ mice. Compared with the Control group, both CIH exposure and Tert^−/−^ deficiency reduced EF and FS. The CIH + Tert^−/−^ group showed the most pronounced systolic impairment; compared with the CIH group, EF and FS were reduced by an additional 24.6 and 15.8 percentage points, respectively (Figure 2A). The HW/BW was also higher in the CIH + Tert^−/−^ group than in the other groups (Figure 2B). H&E staining showed myocardial fiber disarray after CIH exposure and in Tert^−/−^ mice, with more pronounced structural abnormalities in the CIH + Tert^−/−^ group. Representative Masson trichrome staining suggested more prominent interstitial collagen staining in CIH + Tert^−/−^ hearts (Figure 2C). Consistent with these functional and structural abnormalities, serum CK and LDH levels and myocardial BNP expression were further increased in CIH + Tert^−/−^ mice (Figure S1A,B). These findings suggest that CIH exposure further aggravated structural abnormalities, systolic impairment, and myocardial injury markers in the Tert^−/−^ background.

Given that CIH‐induced cardiac remodeling was accompanied by oxidative stress, DNA damage response activation, and upregulation of senescence‐associated markers, and because Tert deficiency is closely linked to telomere dysfunction and DNA damage response activation, we next examined ROS generation, *γ*‐H2AX accumulation, and senescence‐associated marker expression in cardiac tissues from the four groups. DHE staining showed increased myocardial ROS levels in both the CIH and Tert^−/−^ groups compared with the Control group, with a further increase in the CIH + Tert^−/−^ group (Figure 2D). *γ*‐H2AX immunofluorescence showed that both CIH exposure and Tert deficiency increased the percentage of *γ*‐H2AX‐positive nuclei, which was further increased in the CIH + Tert^−/−^ group, indicating enhanced activation of a DNA damage response (Figure 2E). YDGAL fluorescence showed higher SA‐*β*‐gal activity in CIH + Tert^−/−^ myocardium than in either the CIH or Tert^−/−^ group (Figure 2F). Western blot analysis showed a similar pattern, with increased *p*21 and *p*16 expression in the CIH and Tert^−/−^ groups and higher levels in the CIH + Tert^−/−^ group (Figure 2G).

To determine whether these senescence‐related signals were localized to cardiomyocytes, we performed co‐staining of cTnT with *p*21 or *p*16. Compared with the Control group, the proportion of cTnT‐positive cardiomyocytes expressing p21 or p16 was increased in the CIH and Tert^−/−^ groups, and was further increased in the CIH + Tert^−/−^ group (Figure 2H). These results indicate that the telomere dysfunction background represented by Tert deficiency enhances CIH‐associated senescence‐related injury in cardiomyocytes. These findings are consistent with the concept that a senescence‐prone background may reduce myocardial tolerance to intermittent hypoxic stress.

### 3.3 CIH Aggravates D‐Gal‐Induced Senescence‐Associated Phenotypes in Primary Cardiomyocytes

**FIGURE 3:**
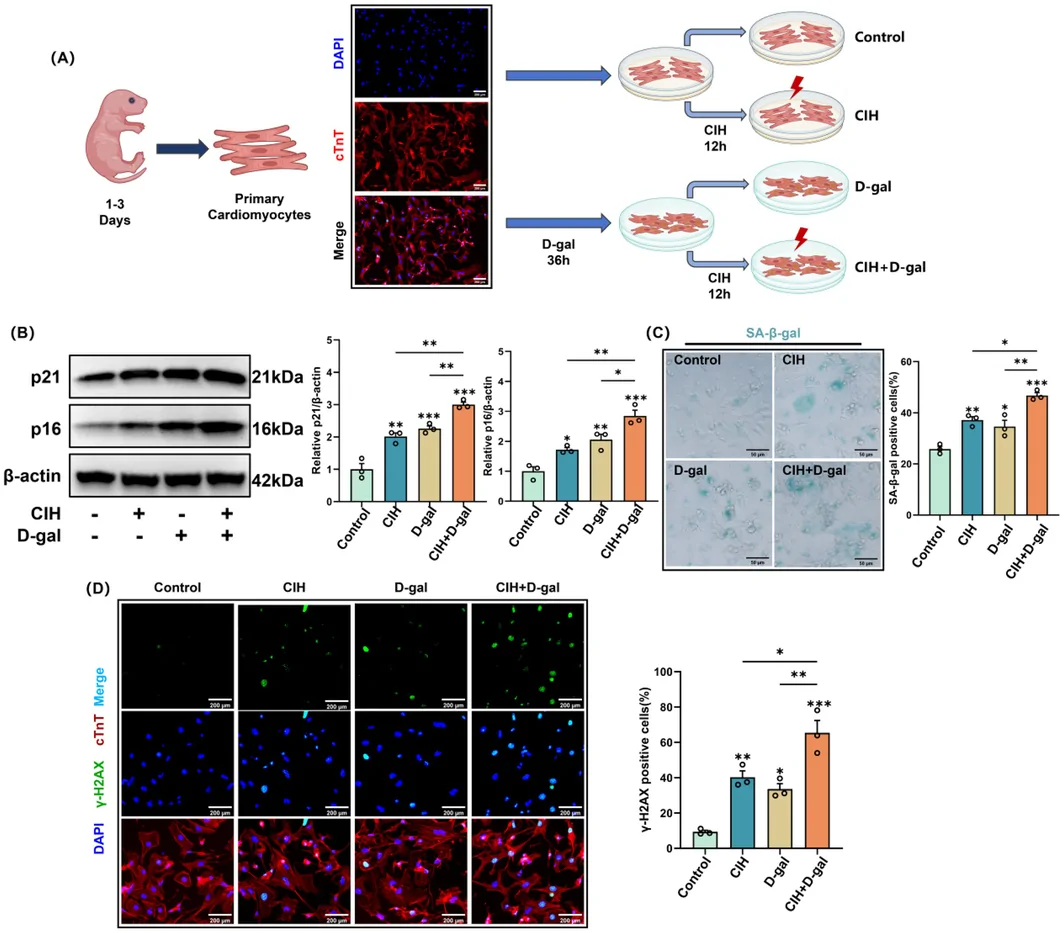
CIH aggravates D‐gal‐induced senescence‐associated phenotypes in primary cardiomyocytes. (A) Schematic showing isolation of neonatal C57BL/6J mouse primary cardiomyocytes, cTnT‐based purity assessment, and assignment to Control, CIH, D‐gal and CIH + D‐gal groups. CIH exposure was performed for 12 h, and total D‐gal treatment lasted 48 h. (B) Representative Western blots and quantitative analysis of *p*21 and *p*16 protein expression in primary cardiomyocytes. *β*‐Actin was used as the loading control. (C) Representative SA‐*β*‐gal staining (Scale bar = 50 μm) and quantification of SA‐*β*‐gal‐positive cells. (D) Representative co‐staining images of *γ*‐H2AX with cTnT, and quantification of *γ*‐H2AX‐positive cardiomyocytes (Green, *γ*‐H2AX; Red, cTnT; Blue, DAPI; Scale bar = 200 μm). For CIH + D‐gal versus CIH and D‐gal, respectively, the adjusted *p* values were 0.002 and 0.010 for *p*21, 0.004 and 0.030 for *p*16, 0.010 and 0.003 for SA‐*β*‐gal, and 0.010 and 0.003 for *γ*‐H2AX. For all panels, quantitative data are presented as mean ± SEM (*n* = 3/group), one‐way ANOVA, *p < 0.05, **p < 0.01, ***p < 0.001.

To further validate the in vivo findings, we established an in vitro model using neonatal C57BL/6J mouse primary cardiomyocytes. Primary cardiomyocytes were assigned to four groups: Control, CIH, D‐gal, and CIH + D‐gal (Figure 3A). Western blot analysis showed that either CIH or D‐gal alone increased p21 and p16 expression, while the CIH + D‐gal group showed further upregulation of both proteins (Figure 3B). SA‐*β*‐gal staining showed an increased proportion of positive cells in the CIH and D‐gal groups compared with the Control group, with a further increase in the CIH + D‐gal group (Figure 3C). Co‐staining of *γ*‐H2AX with cTnT showed that CIH and D‐gal both increased the proportion of *γ*‐H2AX‐positive cardiomyocytes, with a greater increase after combined treatment (Figure 3D). These results indicate that in primary cardiomyocytes, the D‐gal‐induced senescence‐like background enhances CIH‐associated senescence phenotypes and DNA damage response activation.

### 3.4 CIH Aggravates Mitochondrial Dysfunction in D‐Gal‐Induced Senescence‐Like Primary Cardiomyocytes

**FIGURE 4:**
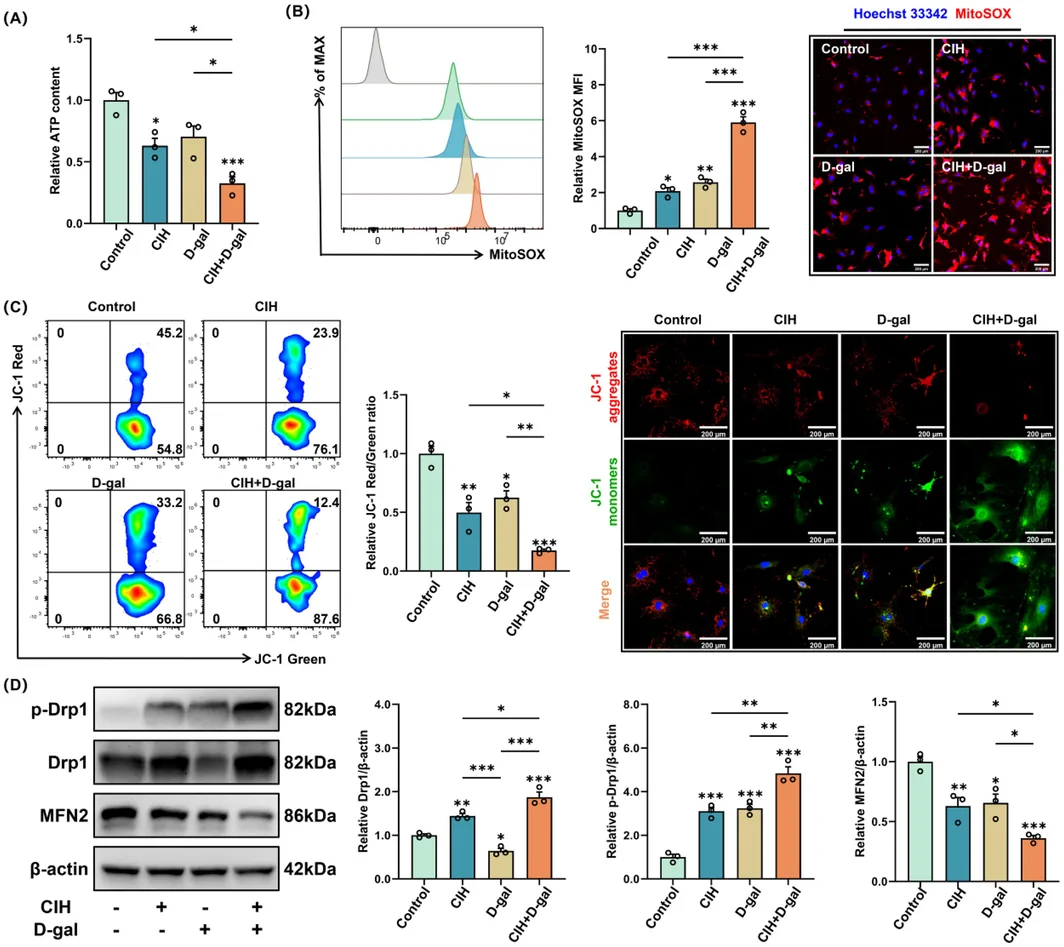
CIH aggravates mitochondrial dysfunction in D‐gal‐induced senescence‐like primary cardiomyocytes. (A) Quantification of ATP content in primary cardiomyocytes. (B) Representative MitoSOX flow cytometry histograms and quantification of relative MitoSOX MFI, together with representative MitoSOX fluorescence images (Red, MitoSOX; Blue, Hoechst 33342; Scale bar = 200 μm). (C) Representative JC‐1 flow cytometry plots and quantification of the JC‐1 red/green ratio, together with representative JC‐1 fluorescence images (Red, JC‐1 aggregates; Green, JC‐1 monomers; Blue, DAPI; Scale bar = 200 μm). (D) Representative Western blots and quantitative analysis of *p*‐Drp1, Drp1 and MFN2 protein expression in primary cardiomyocytes. *β*‐actin was used as the loading control. For CIH + D‐gal versus CIH and D‐gal, respectively, the adjusted *p* values were 0.047 and 0.020 for ATP content, < 0.001 and < 0.001 for MitoSOX MFI, 0.020 and 0.004 for the JC‐1 red/green ratio, 0.010 and < 0.001 for Drp1, 0.001 and 0.003 for *p*‐Drp1, and 0.040 and 0.020 for MFN2. ATP values, MitoSOX MFI, JC‐1 red/green ratio and Western blot densitometry values were normalized to the corresponding control group. *n* = 3 in each experimental group. Data are presented as mean ± SEM and analyzed by one‐way ANOVA. *p < 0.05, **p < 0.01, ***p < 0.001.

Given that CIH enhanced senescence‐associated injury in the D‐gal‐induced senescence‐like background, and that cardiomyocytes depend heavily on mitochondrial energy metabolism and redox homeostasis, we next examined mitochondrial function. ATP analysis showed that CIH or D‐gal alone reduced cellular ATP content, with a greater reduction in the CIH + D‐gal group (Figure 4A). MitoSOX flow cytometry and fluorescence staining showed increased mitochondrial ROS levels after CIH or D‐gal treatment, with a further increase after combined treatment (Figure 4B). JC‐1 analysis showed that CIH and D‐gal decreased the JC‐1 red/green ratio, indicating loss of mitochondrial membrane potential, and the decrease was greater in the CIH + D‐gal group (Figure 4C). Because mitochondrial dynamics contribute to the maintenance of mitochondrial function and redox homeostasis, we further examined proteins related to mitochondrial fission and fusion. Western blot analysis showed that Drp1 and *p*‐Drp1 expression were increased in the CIH + D‐gal group, whereas Mitofusin 2 (MFN2) expression was reduced (Figure 4D). These results suggest that the D‐gal‐induced senescence‐like background amplifies CIH‐associated mitochondrial dysfunction, accompanied by changes in mitochondrial dynamics‐related proteins.

### 3.5 Drp1 Knockdown Partially Attenuates CIH‐Induced Mitochondrial Dysfunction in Senescence‐Like Primary Cardiomyocytes

**FIGURE 5:**
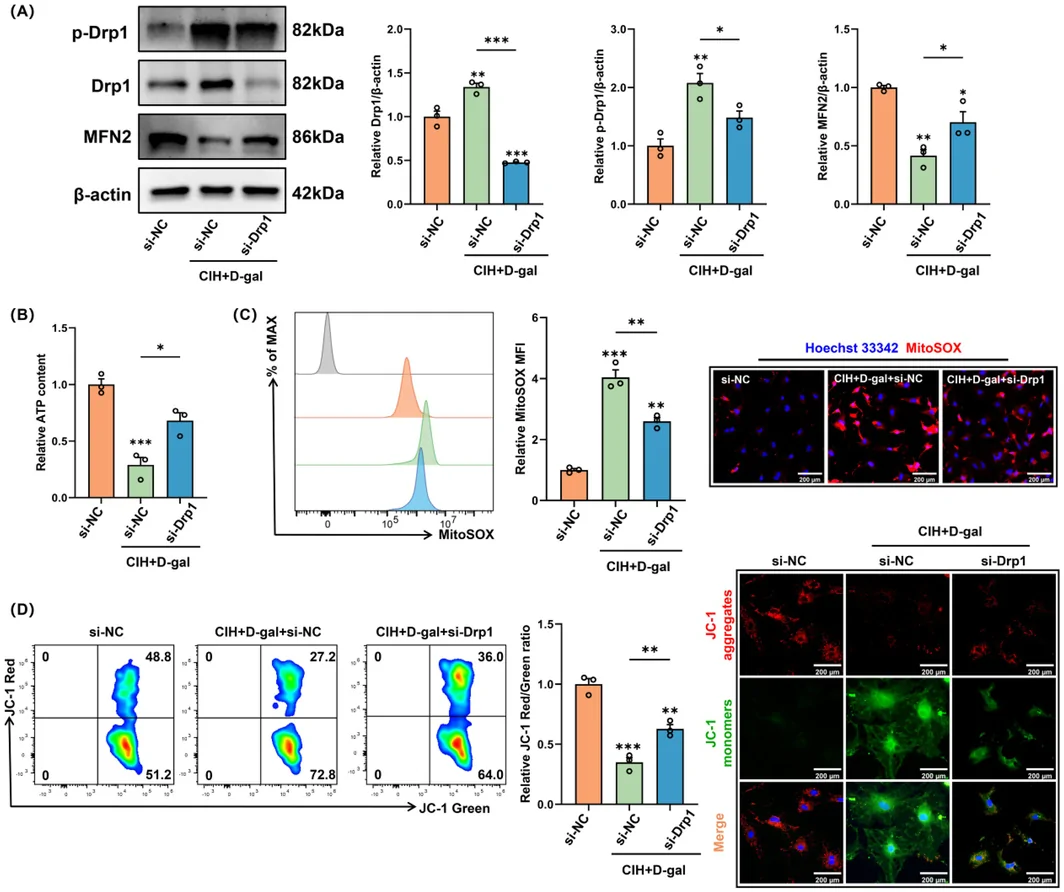
Drp1 Knockdown partially attenuates CIH‐induced mitochondrial dysfunction in senescence‐like primary cardiomyocytes. (A) Representative Western blots and quantitative analysis of *p*‐Drp1, Drp1 and MFN2 protein expression in primary cardiomyocytes from the si‐NC, CIH + D‐gal + si‐NC and CIH + D‐gal + si‐Drp1 groups. *β*‐Actin was used as the loading control. (B) Quantification of ATP content in the three groups of primary cardiomyocytes. (C) Representative MitoSOX flow cytometry histograms and quantification of relative MitoSOX MFI, together with representative MitoSOX fluorescence images (Red, MitoSOX; Blue, Hoechst 33,342; Scale bar = 200 μm). (D) Representative JC‐1 flow cytometry plots and quantification of the JC‐1 red/green ratio, together with representative JC‐1 fluorescence images (Red, JC‐1 aggregates; Green, JC‐1 monomers; Blue, DAPI; Scale bar = 200 μm). For CIH + D‐gal + si‐NC versus CIH + D‐gal+si‐Drp1, the adjusted *p* values were 0.040 for *p*‐Drp1, < 0.001 for Drp1, 0.040 for MFN2, 0.010 for ATP content, 0.002 for MitoSOX MFI, and 0.007 for the JC‐1 red/green ratio. All panels were normalized to the si‐NC group. *n* = 3 in each experimental group. Data are presented as mean ± SEM and analyzed by one‐way ANOVA. *p < 0.05, **p < 0.01, ***p < 0.001.

Because CIH exposure in the D‐gal‐induced senescence‐like background was accompanied by increased Drp1 and p‐Drp1 expression, we next used si‐Drp1 in primary cardiomyocytes to assess whether Drp1 was involved in the mitochondrial vulnerability of senescence‐like cardiomyocytes to CIH. siRNA‐mediated Drp1 knockdown efficiency was confirmed by Western blot before rescue experiments (Figure S2B). In the rescue experiments, compared with the si‐NC group, the CIH + D‐gal + si‐NC group showed increased Drp1 and p‐Drp1 expression and reduced MFN2 expression. si‐Drp1 treatment reduced Drp1 and p‐Drp1 expression and partially restored MFN2 expression (Figure 5A).

We then examined the effect of Drp1 knockdown on mitochondrial function in senescence‐like cardiomyocytes. Cellular ATP content was reduced in the CIH + D‐gal+si‐NC group relative to si‐NC controls; si‐Drp1 treatment restored 55.1% of this deficit (Figure 5B). MitoSOX flow cytometry showed elevated mitochondrial ROS in the CIH + D‐gal + si‐NC group; si‐Drp1 reduced the excess by 47.4%, and fluorescence staining confirmed a consistent pattern (Figure 5C). JC‐1 flow cytometry showed a reduced red/green ratio in the CIH + D‐gal + si‐NC group, indicating mitochondrial membrane potential loss; si‐Drp1 partially restored this ratio, corroborated by JC‐1 fluorescence (Figure 5D). These results support a functional contribution of Drp1 to the enhanced mitochondrial vulnerability of senescence‐like cardiomyocytes under CIH.

### 3.6 Drp1 Knockdown Attenuates CIH‐Induced Senescence‐Associated Phenotypes in Senescence‐Like Primary Cardiomyocytes

**FIGURE 6:**
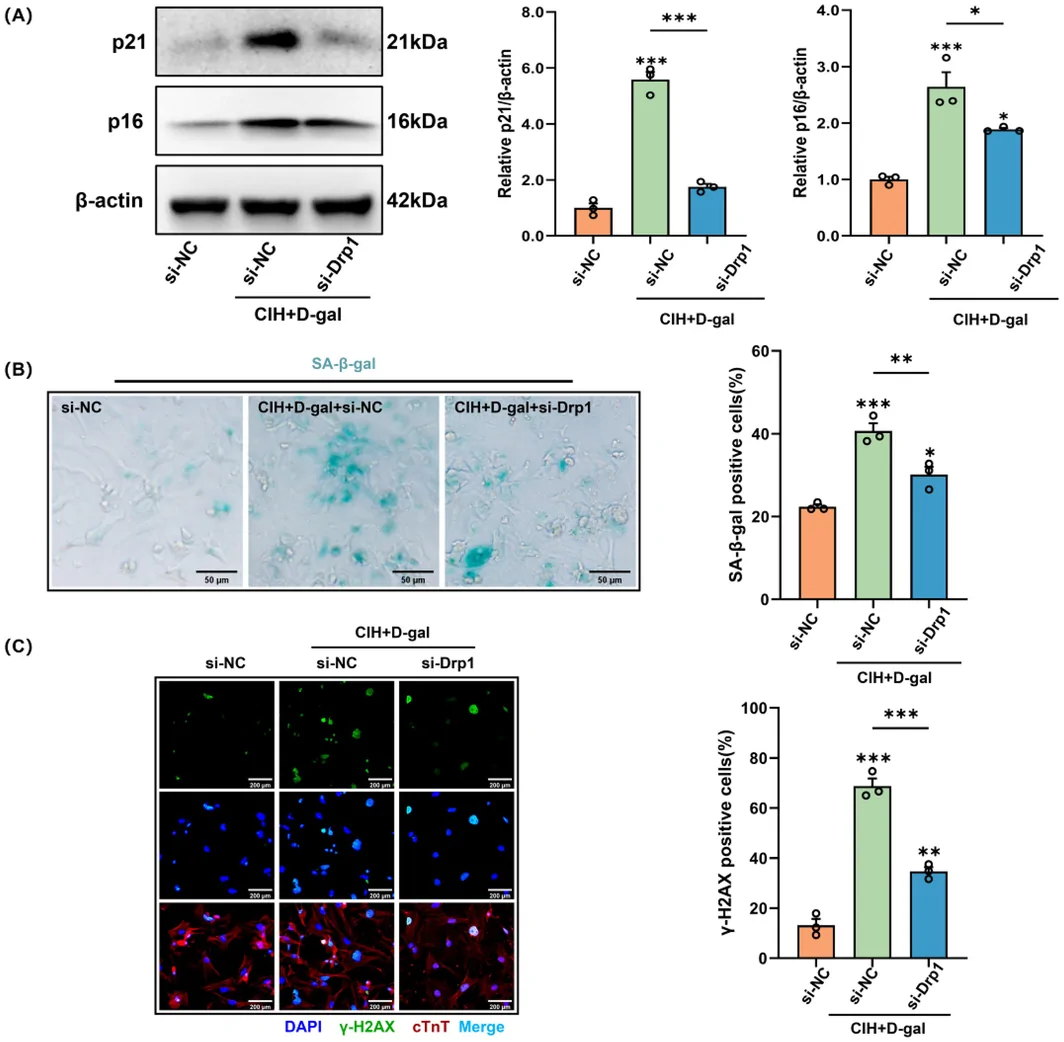
Drp1 knockdown attenuated CIH‐induced senescence‐associated phenotypes in senescence‐like primary cardiomyocytes. (A) Representative immunoblots and quantification of *p*21 and *p*16 in si‐NC, CIH + D‐gal + si‐NC and CIH + D‐gal + si‐Drp1 groups. (B) SA‐*β*‐gal staining and quantification of SA‐*β*‐gal‐positive cells (Scale bar = 50 μm). (C) *γ*‐H2AX and cTnT co‐staining and quantification of *γ*‐H2AX‐positive cardiomyocytes (Green, *γ*‐H2AX; Red, cTnT; Blue, DAPI; Scale bar = 200 μm). For CIH + D‐gal+si‐NC versus CIH + D‐gal+si‐Drp1, the adjusted *p* values were < 0.001 for *p*21, 0.030 for *p*16, 0.007 for SA‐*β*‐gal, and < 0.001 for *γ*‐H2AX. Western blot densitometry values were normalized to the si‐NC group. *n* = 3 in each experimental group. Data are presented as mean ± SEM and analyzed by one‐way ANOVA. *p < 0.05, **p < 0.01, ***p < 0.001.

After observing that Drp1 knockdown partially improved CIH‐induced mitochondrial dysfunction in senescence‐like primary cardiomyocytes, we further examined senescence‐associated markers and DNA damage response activation. Western blot analysis showed that *p*21 and *p*16 expression were increased in the CIH + D‐gal+si‐NC group compared with the si‐NC group. si‐Drp1 treatment reduced *p*21 expression and partially reduced p16 expression (Figure 6A). SA‐*β*‐gal staining showed an increased proportion of positive cells in the CIH + D‐gal + si‐NC group, whereas si‐Drp1 treatment reduced the proportion of SA‐*β*‐gal‐positive cells (Figure 6B). Co‐staining of *γ*‐H2AX with cTnT further showed that the proportion of *γ*‐H2AX‐positive cardiomyocytes was increased in the CIH + D‐gal+si‐NC group and reduced after si‐Drp1 treatment (Figure 6C). These results indicate that Drp1 knockdown attenuates CIH‐induced senescence‐associated phenotypes and DNA damage response activation in senescence‐like primary cardiomyocytes.

## 4 Discussion

The present study was designed to determine whether a pre‐existing aging‐related myocardial susceptibility modifies the cardiac response to CIH, and to identify potential mechanistic points of convergence between CIH‐induced stress and senescence‐like cardiomyocyte vulnerability. To this end, we adopted a cross‐model strategy combining an in vivo telomere dysfunction‐associated model, Tert^−/−^ mice, with an in vitro D‐gal induced senescence‐like primary cardiomyocyte model. These two models differ in their upstream biological mechanisms but share aging‐related molecular features relevant to stress susceptibility. Both should be interpreted as experimental susceptibility models rather than as models of natural or physiological aging. The principal findings were that CIH initiated early adverse myocardial remodeling in young wild‐type mice and was accompanied by activation of senescence‐associated molecular signals; both susceptibility models further amplified this response; and siRNA‐mediated Drp1 knockdown partially improved CIH‐induced mitochondrial dysfunction in senescence‐like cardiomyocytes while concomitantly attenuating DNA damage response activation and senescence‐associated phenotypes. Taken together, these findings suggest that Drp1 may contribute functionally to the amplification of CIH‐induced cardiomyocyte injury in the setting of aging‐related myocardial susceptibility, but the present evidence does not establish Drp1 as the principal mechanistic driver.

In young wild‐type mice, EF and FS showed a progressive but modest decline during CIH exposure, while overall systolic function remained relatively preserved. This phenotype is more consistent with an early remodeling stage than with overt systolic decompensation, in line with previous rodent studies using comparable intermittent hypoxia exposure protocols (Bourdier et al. 2020; Arnaud et al. 2023). The functional, structural, and molecular evidence collectively indicate that repeated hypoxia‐reoxygenation cycles are sufficient to initiate early myocardial injury in the young heart, but do not constitute an overt heart failure phenotype. In parallel, in vivo DHE staining showed increased myocardial ROS levels, accompanied by a higher proportion of *γ*‐H2AX‐positive nuclei, increased SA‐*β*‐gal activity, and *p*21/*p*16 protein upregulation. These changes are consistent with established links among oxidative stress, DNA damage response activation, and senescence‐associated signaling (Mehdizadeh et al. 2021). It should be noted, however, that SA‐*β*‐gal positivity, *γ*‐H2AX accumulation, and *p*21/*p*16 induction are not specific to stable senescence and may also reflect acute stress responses or injury signaling. Accordingly, these findings are interpreted as senescence‐associated molecular changes rather than direct evidence of stable, irreversible cellular senescence.

Tert deficiency represents a genetically defined susceptibility model associated with telomere dysfunction. Previous studies have shown that telomere shortening can activate DNA damage responses and contribute to cardiovascular remodeling (Chatterjee et al. 2024; Leri et al. 2003; Wang et al. 2026). In contrast, D‐gal induces a senescence‐like stress state in primary cardiomyocytes mainly through mitochondrial ROS overproduction and oxidative macromolecular damage, thereby partially recapitulating redox and metabolic abnormalities associated with cellular aging (Wang et al. 2022; Peng et al. 2025; Liu et al. 2024). In this study, both the in vivo Tert^−/−^ model and the in vitro D‐gal model showed that combined exposure to CIH and an aging‐related background caused more severe injury than either condition alone, involving cardiac function, myocardial injury, mitochondrial function, DNA damage response activation, and senescence‐associated phenotypes. It should be emphasized that these two models differ in their upstream mechanisms and neither fully recapitulates the systemic complexity of natural aging, including immune, endocrine, vascular, and intercellular paracrine changes. Nevertheless, the consistent direction of the findings across two mechanistically distinct models supports the view that aging‐related susceptibility lowers the myocardial threshold for CIH‐induced maladaptive remodeling. Co‐staining of cTnT with *p*21, *p*16, and *γ*‐H2AX further localized the enhanced senescence‐associated signals to cardiomyocytes, indicating that cardiomyocytes are an important cellular source of this amplified response, consistent with prior evidence linking cardiomyocytes positive for senescence markers to adverse cardiac remodeling (Grootaert 2024; Redgrave et al. 2023).

Cardiomyocytes are post‐mitotic cells with high dependence on mitochondrial oxidative phosphorylation for contractile energy supply and on mitochondrial redox buffering for cellular homeostasis (Lopaschuk et al. 2021; Murphy et al. 2016). Mitochondrial dysfunction may therefore represent a key point of convergence between CIH‐induced stress and aging‐related cellular vulnerability. In the present study, the D‐gal induced‐senescence‐like state increased the susceptibility of primary cardiomyocytes to CIH‐induced mitochondrial injury, as shown by reduced ATP levels, increased mitochondrial superoxide generation detected by MitoSOX, and greater loss of mitochondrial membrane potential detected by JC‐1. These functional abnormalities were accompanied by coordinated changes in mitochondrial dynamics markers, including increased Drp1 and p‐Drp1 (Ser616) and reduced MFN2. Drp1‐mediated mitochondrial fission has been implicated in cardiomyocyte injury under ischemia–reperfusion, pressure overload, oxidative stress, and metabolic stress (Piao et al. 2024; Li et al. 2022; Disatnik et al. 2013; Shirakabe et al. 2016). Excessive fission may promote the accumulation of fragmented and depolarized mitochondria, impair electron transport efficiency, increase mitochondrial ROS production (Niu et al. 2024), and thereby reinforce DNA damage response activation and senescence‐associated signaling (Miwa et al. 2022). In this context, the concomitant increase in Drp1/p‐Drp1 and decrease in MFN2 under CIH and D‐gal co‐treatment were consistent with a fission‐biased mitochondrial dynamics marker profile that may contribute to the amplified vulnerability of senescence‐like cardiomyocytes to CIH. Drp1 knockdown restored 55.1% of the ATP deficit and reduced 47.4% of the mitochondrial ROS excess, while only partially restoring membrane potential, supporting a contributory rather than dominant role. However, these intervention data do not resolve the temporal and causal relationships linking mitochondrial alterations to DNA damage response activation and senescence‐associated phenotypes.

From a translational perspective, our findings suggest that the cardiac impact of OSA‐related CIH may depend not only on hypoxic burden itself, but also on the biological state of the target myocardium. Telomere dysfunction or a senescence‐like background may lower myocardial tolerance to repeated hypoxia‐reoxygenation, thereby facilitating the shift from compensated adaptation to maladaptive remodeling. Integrating indices of hypoxic burden with biomarkers related to cellular aging, oxidative stress, or mitochondrial stress may therefore help identify OSA patients at higher risk of cardiac injury. This translational hypothesis requires further validation in human studies.

Several limitations should be acknowledged. First, naturally aged animals were not included. Although Tert^−/−^ mice and D‐gal treatment provide two aging‐related susceptibility backgrounds, neither model fully recapitulates the systemic complexity of chronological aging. Future studies incorporating naturally aged animals will be needed to directly validate the effect of chronological aging on CIH tolerance. In addition, telomere length and telomere‐associated DNA damage were not directly assessed; therefore, Tert^−/−^ should be interpreted as a susceptibility background related to telomere dysfunction rather than as direct evidence that telomere attrition mediates CIH‐induced injury. Second, only female mice were used, and WT controls were not littermates of Tert^−/−^ mice, so sex‐specific responses and background‐related variability cannot be completely excluded. Third, senescence was inferred from multiple associated markers, including SA‐*β*‐gal activity, *γ*‐H2AX accumulation, and *p*21/*p*16 upregulation, rather than from direct evidence of stable cell‐cycle arrest or irreversible senescence. Future studies should include direct evidence of stable growth arrest and broader SASP profiling to more rigorously define cardiomyocyte senescence under CIH. Fourth, mitochondrial characterization was limited to ATP content, MitoSOX fluorescence, JC‐1 membrane potential, and selected dynamics‐related proteins. These surrogate readouts do not directly define mitochondrial respiratory capacity or mitochondrial quality‐control function. Mitochondrial respiration, fragmentation, Drp1 mitochondrial translocation, mitophagic flux, upstream signaling, and metabolic tracing were not assessed, limiting characterization of the nature and extent of the observed mitochondrial dysfunction. Finally, the in vitro experiments were performed in neonatal primary cardiomyocytes, and Drp1 knockdown was tested only in this cellular system. Future studies using adult cardiomyocytes, in vivo genetic modulation of Drp1, and cell type‐specific analyses will be needed to further define the contribution of cardiomyocytes and non‐cardiomyocyte populations to CIH‐induced cardiac remodeling.

## Author Contributions

Conceptualization and Design: Yinghui Su, Yuyang Miao, and Jin Tan; Experimental Execution (Animal and Cell Culture): Yinghui Su and Yuyang Miao; Data Interpretation and Analysis: Jin Tan and Feng Wang; Manuscript Drafting and Refinement: Yinghui Su, Yuyang Miao, and Qiang Zhang; Final Approval: All authors have read, commented on, and approved the final version of the manuscript.

## Funding

Qiang Zhang received grants from the National Natural Science Foundation of China [Grant No. U25A2002], the National Key Research and Development Program of China [Grant No. 2023YFC3605200], and Tianjin Key Medical Discipline Construction Project [Grant No. TJYXZDXK‐3‐005C].

## Conflicts of Interest

The authors declare no conflicts of interest.

## Supporting information


**Figure S1:** Supportive myocardial injury readouts after CIH exposure in the telomere dysfunction background. Mice were studied in four groups: Control, CIH, Tert^−/−^ and CIH + Tert^−/−^, with all analyses performed after 8 weeks of CIH exposure. (A) Serum CK and LDH levels in the four groups. (B) Representative Western blots and quantitative analysis of BNP protein expression in cardiac tissue. *β*‐Tubulin was used as the loading control. Western blot densitometry was normalized to the corresponding control group. Data are presented as mean ± SEM and analyzed using one‐way ANOVA (*n* = 3/group). Each dot represents one mouse or one biological replicate. *p < 0.05, **p < 0.01, ***p < 0.001.
**Figure S2:** Purity assessment of primary cardiomyocytes and validation of Drp1 siRNA knockdown efficiency. (A) Representative cTnT immunofluorescence images and quantification of cardiomyocyte purity from three independent primary cardiomyocyte isolations (Red, cTnT; Blue, DAPI; Scale bar = 200 μm). Cardiomyocyte purity was calculated as the percentage of cTnT positive cells among total DAPI‐positive cells. Three randomly selected fields were analyzed for each independent isolation, and each dot represents one field. (B) Representative Western blots and quantitative analysis of Drp1 protein expression after transfection with si‐NC, siRNA‐1 or siRNA‐2. *β*‐actin was used as the loading control. Western blot densitometry values were normalized to the si‐NC group. For panel B, data are presented as mean ± SEM (*n* = 3 in each experimental group, one‐way ANOVA). ***p < 0.001.
**Table S1:** Critical reagents and commercial assays.
**Table S2:** A list of abbreviations.

## Data Availability

The data that support the findings of this study are available on request from the corresponding author. The data are not publicly available due to privacy or ethical restrictions.
