# Supplementary material for: Aging‐Related Myocardial Susceptibility Lowers Cardiac Tolerance to Chronic Intermittent Hypoxia Through Dynamin‐Related Protein 1‐Associated Mitochondrial Vulnerability
Source: Aging Cell. 2026 Aug 25;25(9):e70684. doi: 10.1111/acel.70684 (PMC13507029; doi:10.1111/acel.70684)
Supplement: Supplementary file 1 [file ACEL-25-e70684-s001.docx]

**Aging-related Myocardial Susceptibility Lowers Cardiac Tolerance to Chronic Intermittent Hypoxia through Dynamin-related Protein 1-associated Mitochondrial Vulnerability**

Yinghui Su^1†^, Yuyang Miao^1†^, Jin Tan ^1^, Feng Wang ^2^, Qiang Zhang^1*^

**Figure S1. Supportive myocardial injury readouts after CIH exposure in the telomere dysfunction background.** Mice were studied in four groups: Control, CIH, Tert^-/-^ and CIH+Tert^-/-^, with all analyses performed after 8 weeks of CIH exposure. **(A)** Serum CK and LDH levels in the four groups. **(B)** Representative Western blots and quantitative analysis of BNP protein expression in cardiac tissue. β-Tubulin was used as the loading control. Western blot densitometry was normalized to the corresponding control group. Data are presented as mean ± SEM and analyzed using one-way ANOVA (n = 3/group). Each dot represents one mouse or one biological replicate. *P < 0.05, **P < 0.01, ***P < 0.001.

**Figure S2. Purity assessment of primary cardiomyocytes and validation of Drp1 siRNA knockdown efficiency. (A)** Representative cTnT immunofluorescence images and quantification of cardiomyocyte purity from three independent primary cardiomyocyte isolations (Red, cTnT; Blue, DAPI; Scale bar = 200 μm). Cardiomyocyte purity was calculated as the percentage of cTnT positive cells among total DAPI-positive cells. Three randomly selected fields were analyzed for each independent isolation, and each dot represents one field. **(B)** Representative Western blots and quantitative analysis of Drp1 protein expression after transfection with si-NC, siRNA-1 or siRNA-2. β-actin was used as the loading control. Western blot densitometry values were normalized to the si-NC group. For panel B, data are presented as mean ± SEM (n = 3 in each experimental group, one-way ANOVA). ***P < 0.001.

**Supplementary Table S1.** Critical reagents and commercial assays

| **Product Name** | **Vendor** | **Cat. No.** | **Applications** |
| --- | --- | --- | --- |
| cTnT | HuanBio | EM1701-39 | IF |
| p21 | Proteintech | 28248-1-AP | WB, IF |
| p16 | Abmart | TA5484F | WB, IF |
| γ-H2AX | Beyotime | C2035S | IF |
| Drp1 | Santa Cruz | sc-271583 | WB |
| p-Drp1 | Cell Signaling Technology | 3455S | WB |
| MFN2 | ABclonal | A19678 | WB |
| BNP | Wanleibio | WL02126 | WB |
| β-actin | Cell Signaling Technology | 4967S | WB |
| β-Tubulin | Wanleibio | WL01931 | WB |

**Supplementary Table S2.** A List of Abbreviations

| **Abbreviations** | **Full term** |
| --- | --- |
| OSA | obstructive sleep apnea |
| CIH | chronic intermittent hypoxia |
| Drp1 | Dynamin-related protein 1 |
| MFN2 | Mitofusin 2 |
| Tert^-/-^ | telomerase reverse transcriptase-deficient |
| D-gal | D-galactose |
| HW/BW | heart weight to body weight ratio |
| CK | creatine kinase |
| LDH | lactate dehydrogenase |
| EF | ejection fraction |
| FS | fractional shortening |
| DHE | dihydroethidium |
| SA-β-gal | senescence-associated beta-galactosidase |
| SEM | standard error of the mean |
